# Growth Differentiation Factor-11 Causes Neurotoxicity During Ischemia *in vitro*

**DOI:** 10.3389/fneur.2020.01023

**Published:** 2020-09-10

**Authors:** Brad A. Sutherland, Gina Hadley, Zoi Alexopoulou, Tiffany A. Lodge, Ain A. Neuhaus, Yvonne Couch, Nareg Kalajian, Karl J. Morten, Alastair M. Buchan

**Affiliations:** ^1^Acute Stroke Programme, Radcliffe Department of Medicine, University of Oxford, Oxford, United Kingdom; ^2^Tasmanian School of Medicine, College of Health and Medicine, University of Tasmania, Hobart, TAS, Australia; ^3^Nuffield Department of Clinical Neurosciences, University of Oxford, Oxford, United Kingdom; ^4^Nuffield Department of Womens and Reproductive Health, University of Oxford, Oxford, United Kingdom

**Keywords:** growth factor, GDF-11, stroke, neuron, ischemia, *in vitro*

## Abstract

Age-related neuronal dysfunction can be overcome by circulating factors present in young blood. Growth differentiation factor-11 (GDF-11), a systemic factor that declines with age, can reverse age-related dysfunction in brain, heart and skeletal muscle. Given that age increases susceptibility to stroke, we hypothesized that GDF-11 may be directly protective to neurons following ischemia. Primary cortical neurons were isolated from E18 Wistar rat embryos and cultured for 7–10 days. Neurons were deprived of oxygen and glucose (OGD) to simulate ischemia. Neuronal death was assessed by lactate dehydrogenase, propidium iodide or CellTox™ green cytotoxicity assays. 40 ng/mL GDF-11 administration during 2 h OGD significantly increased neuronal death following 24 h recovery. However, GDF-11 pre-treatment did not affect neuronal death during 2 h OGD. GDF-11 treatment during the 24 h recovery period after 2 h OGD also did not alter death. Real-time monitoring for 24 h revealed that by 2 h OGD, GDF-11 treatment had increased neuronal death which remained raised at 24 h. Co-treatment of 1 μM SB431542 (ALK4/5/7 receptor inhibitor) with GDF-11 prevented GDF-11 neurotoxicity after 2 h OGD and 24 h OGD. Transforming growth factor beta (TGFβ) did not increase neuronal death to the same extent as GDF-11 following OGD. GDF-11 neurotoxicity was also exhibited following neuronal exposure to hydrogen peroxide. These results reveal for the first time that GDF-11 is neurotoxic to primary neurons in the acute phase of simulated stroke through primarily ALK4 receptor signaling.

## Introduction

The increased life expectancy of twenty-first century living has led to a substantial rise in the number of age-related disorders including stroke. Even in the thrombectomy era where a number of ischemic stroke patients are receiving reperfusion therapy (either pharmacologically with alteplase or mechanically via thrombectomy), the majority of ischemic stroke patients are left with no therapeutic options acutely to limit the damage of ischemia (1). The search continues for both primary and adjunctive neuroprotective therapies that can protect the brain from the resulting injury following an ischemic stroke (2).

Anti-aging factors are hypothesized to slow down the aging process and improve the quality of life of the aged. Recently, it was revealed that age-related neuronal dysfunction can be overcome by circulating factors present in young blood (3). A number of factors exist in young blood that are not found in old blood. One of these factors, considered to be an anti-aging factor is growth differentiation factor-11 (GDF-11) as its levels in blood decline with age (4), though this has been challenged due to problems with detection methods for GDF-11 measurement (5). In addition, systemic (i.p.) administration of recombinant GDF-11 has been shown to reverse age-related dysfunction in the rodent brain (4), heart (6) and skeletal muscle (7). GDF-11 is a growth factor that belongs to the transforming growth factor-beta (TGFβ) family and acts upon a number of TGFβ receptors including ALK4 and ALK5 (8). Previous studies have shown that TGFβ receptor agonists show neuroprotection whereas TGFβ receptor antagonists have exacerbated injury in pre-clinical stroke models (9). Two recent studies have trialed GDF-11 as a neuroprotectant in middle cerebral artery occlusion models, and both showed that recombinant GDF-11 administration reduced neurobehavioural deficits through the augmentation of angiogenesis, endothelial cell proliferation and increased neural precursor proliferation out to 14 days post-stroke (10, 11). Further evidence supporting this was the fact that SB431542, a ALK4/5/7 receptor inhibitor, blocked the protective effects of GDF-11 (10, 11).

Given its effectiveness on the vascular system and the ability of GDF-11 to modulate age-related neuronal dysfunction, we hypothesize that GDF-11 could be an ideal candidate as a neuroprotective agent for ischemic stroke. Therefore, in this study, we sought to determine whether GDF-11 administration to primary neurons could provide direct protection to neurons using an *in vitro* model of ischemia.

## Materials and Methods

To determine the effects of GDF-11 directly on neurons, we used a primary cortical neuronal culture as we have previously described (12, 13). Neurons were plated onto poly-D-lysine-coated 12 well plates at 10^5^ cells per well in complete neurobasal media containing 2% B27 serum-free supplement, 2 mM L-glutamine and 1% penicillin/streptomycin in neurobasal medium (all Invitrogen). Neurons remained in culture for 7–10 days to allow neuronal networks to develop before experiments were conducted.

GDF-11 was purchased from Peprotech (cat#120-11, UK). To determine whether GDF-11 altered basal viability of neurons, GDF-11 was diluted in complete neurobasal media to produce a concentration range of 4–400 ng/mL and added to neuronal cultures for 48 h. Neuronal viability was assessed with a CellTiter 96 assay (Promega, UK) following manufacturer's instructions, with neurons being incubated for 5 h with the reaction reagent.

**Figure 1:**
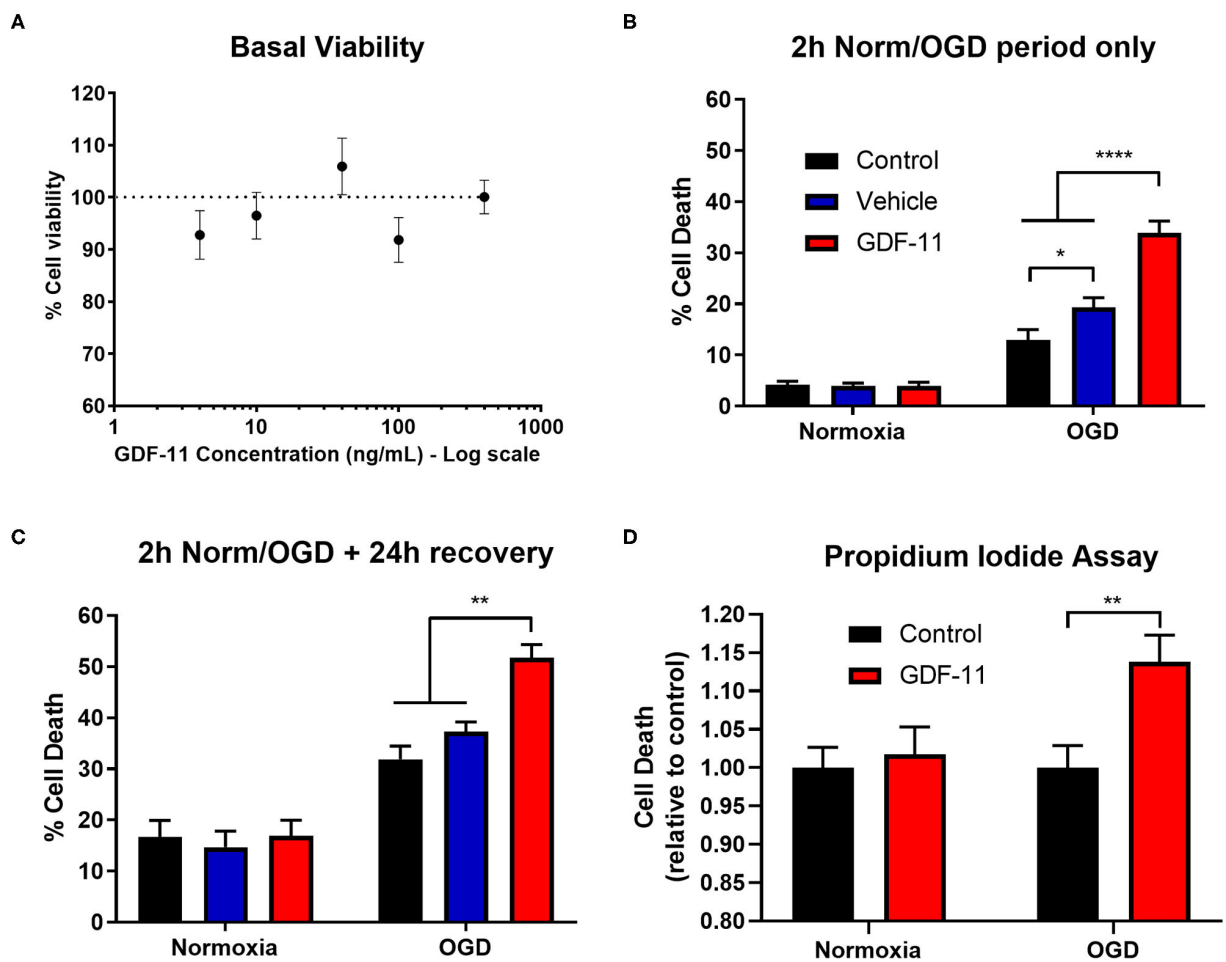
GDF-11 increased neuronal death during OGD. **(A)** Cortical neurons kept under normoxic conditions were exposed to GDF-11 (4–400 ng/mL) for 48 h. Percentage neuronal viability was quantified with the CellTiter 96 assay. N=4 for each condition. 2 h OGD **(B)** and 2 h OGD + 24 h recovery **(C)** increased cortical neuronal death compared to normoxia while GDF-11 increased cell death compared with media and DMSO under OGD conditions. GDF-11 did not affect neuronal death under normoxic conditions. Cell death was quantified by LDH assay. Groups: media (control) (*n* = 6), DMSO (control) (*n* = 6), GDF-11 (*n* = 3). **(D)** GDF-11 increased cell death following 2 h OGD + 24 h recovery, but not under normoxia conditions using a propidium iodide assay. *N* = 8–16 per condition. All data are presented as mean ± SEM. Two-way ANOVA with Tukey's *post-hoc* Test. *p < 0.05, **p < 0.01, ****p < 0.0001.

To determine whether GDF-11 altered neuronal viability under simulated ischemic conditions, we performed OGD experiments. Days *in vitro* (DIV) 7–10 primary cortical neurons were exposed to 0% oxygen using a hypoxic chamber (Coy) coupled with glucose-free neurobasal media (Invitrogen) for 2 h as described previously (12, 13), or using an environmentally-controlled microplate reader (Omega, BMG Labtech) for up to 24 h exposure. Following 2 h OGD in the hypoxic chamber, cultures were returned to normoxic conditions with glucose-containing neurobasal media for 24 h. For all OGD experiments, 40 ng/mL GDF-11 was used due to this being the concentration in which effects in brain capillary endothelial cells have been observed (4) and there was no neurotoxicity under normoxic conditions (Figure 1A). Depending on the experiment, GDF-11 was administered for 7 days prior to OGD, during OGD or for 24 h following OGD in the recovery phase.

To assess neuronal death, a lactate dehydrogenase (LDH) activity assay (Cytotoxicity kit, Promega, UK), propidium iodide assay or CellTox^TM^ green cytotoxicity assay (Promega, UK) was used as per manufacturer's instructions. Briefly, for the LDH assessment, media were collected at the end of the relevant period for measurement of LDH release. The cells were then lysed with 1% Triton X-100 in complete neurobasal medium to obtain total LDH released. To get a percentage cell death, the LDH released during the relevant period was divided by the sum of the LDH released during that period and during cell lysis (12, 13). For the propodium iodide assay, cells were plated in 96-well plates at 50,000 cells/well and following the OGD protocol, propidium iodide (final concentration 50 μM, Invitrogen, UK) was added and incubated for 30 min. Plates were then assessed for the level of cell death as indicated by propidium iodide fluorescence (excitation 540 nm emission 620 nm) using a fluorometer (Omega Optima, BMG Labtech, UK). The CellTox^TM^ green cytotoxicity assay was used per manufacturer's instructions for real-time monitoring of cell death as it measures cell free DNA in the media or DNA of dead cells with a compromised plasma membrane (14). After administration of the CellTox^TM^ Green reagent, and placed in the environmentally controlled plate reader (Omega, BMG Labtech), a gas permeable membrane (Breathe-Easy sealing membrane, Sigma) was used to prevent evaporation while still allowing gas exchange. A concurrent positive control was performed using lysis buffer (Promega).

Neurons possess ALK4 and ALK5 receptors (15, 16), and so to determine whether the effects of GDF-11 were mediated by the ALK4/5 receptors on neurons, we performed neuronal OGD experiments in the presence of GDF-11 with or without 1 μM SB431542 (an ALK4/5/7 receptor inhibitor, Tocris) (17). TGFβ is a ligand for ALK5 receptors (15, 18) and so we performed neuronal OGD experiments with 10 ng/mL TGFβ to determine its effect on neurotoxicity.

During ischemia, neurons were exposed to a number of potentially damaging factors including lack of energy supply, oxidative stress and excitotoxicity. We exposed cortical neuronal cultures to 100 μM iodoacetate (to inhibit glycolysis) and 10 μM antimycin A (to inhibit mitochondrial respiration) as a model of chemical ischemia, 500 μM glutamate as a model of excitotoxicity, and 1 mM hydrogen peroxide as a model of oxidative stress for 24 h. Experiments were carried out in the absence or presence of GDF-11. Neuronal death was determined by LDH activity of the incubating media during the insults.

Data were analyzed by a two way ANOVA followed by a Tukey's *post-hoc* test when there were two independent variables or a one way ANOVA followed by a Tukey's or Bonferroni's *post-hoc* test when there was only one variable. All analyses were performed using GraphPad Prism v7.0. Data are presented as mean + SEM. All results are from at least three experiments, *p* < 0.05 is considered statistically significant. All data and research materials are available upon request.

## Results

### 48 h GDF-11 Administration Did Not Alter Cellular Viability in Primary Cortical Neurons

Initially, we wished to test whether GDF-11 altered neuronal viability under standard culture conditions. Exposure of 4–400 ng/mL GDF-11 to neurons for 48 h did not affect neuronal viability (all concentrations had >90% viability; Figure 1A). Since Katsimpardi et al. had previously shown effects of 40 ng/mL GDF-11 in brain capillary endothelial cells (4), we selected this concentration for subsequent studies.

### Exposure of 40 ng/mL GDF-11 to Neurons During 2 h OGD Exacerbated Neuronal Death

Given the potential neuroprotective effects of GDF-11 (10, 11), we wished to determine whether GDF-11 could provide direct neuroprotection to ischemic neurons. Following 2 h OGD (Figure 1B), there was a significant increase in cell death during OGD compared to normoxia [*F*_(1, 48)_ = 201.7, *p* < 0.0001] as detected by an LDH assay. Administration of 40 ng/mL GDF-11 to neurons during 2 h OGD significantly increased neuronal death (vehicle: 19.3% ± 1.9% cell death, GDF-11: 33.9% ± 2.3%; Tukey's *p* < 0.0001). At 24 h recovery following 2 h OGD (Figure 1C), the OGD group continued to have significantly increased cell death compared to normoxia [*F*_(1, 48)_ = 111.1, *p* < 0.0001], with GDF-11 increasing cell death compared to OGD alone (vehicle: 37.3% ± 1.9% cell death, GDF-11: 51.7% ± 2.6%; Tukey's *p* = 0.0019). This neurotoxicity by GDF-11 following OGD was replicated using a propidium iodide assay (1.14 ± 0.04 fold over control, Tukey's *p* = 0.003; Figure 1D). There was no significant effect of GDF-11 treatment on neuronal death under normoxic conditions (Figure 1).

### GDF-11 Treatment Prior to OGD or After OGD Did Not Alter Neuronal Death

**Figure 2:**
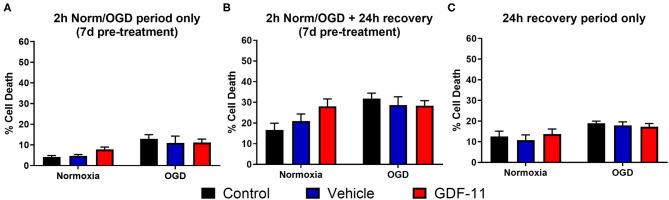
GDF-11 had no effect on neuronal death with treatment prior to OGD and after OGD. **(A)** 7 days pre-treatment with 40 ng/mL GDF-11 had no effect on cortical neuronal death during 2 h normoxia or OGD. GDF-11 was not present during the OGD phase. **(B)** 7 days pre-treatment with 40 ng/mL GDF-11 did not affect cortical neuronal death following 2 h normoxia or OGD and 24 h recovery. GDF-11 was not present during the OGD phase. **(C)** Treatment with GDF-11 during 24 h recovery only did not affect cortical neuronal death following 2 h normoxia or OGD. GDF-11 was not present during the OGD phase. Cell death was quantified by LDH assay. Data presented as mean ± SEM. Groups: control (*n* = 6), DMSO (*n* = 3) and GDF-11 (*n* = 3). Two-way ANOVA with Tukey's *post-hoc* test.

Given that GDF-11 administration to ischemic neurons worsened cell death, we then carried out experiments to determine whether treatment with GDF-11 prior to OGD or after OGD could alter this neurotoxic effect. Again, there was a significant increase in cell death with OGD compared to normoxia [Figure 2A, *F*_(1, 36)_ = 17.0, *p* = 0.0002]. However, 7 days of pre-treatment with GDF-11 did not affect neuronal death during 2 h OGD compared to vehicle pre-treatment (vehicle: 11.0% ± 3.3%, GDF-11: 11.1% ± 1.7%; Tukey's *p* = 0.998). Similar results were observed at 24 h recovery following 2 h OGD (Figure 2B; vehicle: 28.7% ± 4.0%, GDF-11: 28.4% ± 2.4%; Tukey's *p* = 0.998). When measuring cell death during 24 h recovery after 2 h OGD or normoxia (Figure 2C), there was a small but significant increase in neuronal death following OGD [*F*_(1, 48)_ = 11.1, *p* = 0.0017]. When GDF-11 was administered during the 24 h recovery period only following 2 h OGD (Figure 2C), there was no significant effect of GDF-11 treatment on the ability of OGD to kill neurons (vehicle: 17.9% ± 1.7%, GDF-11: 17.2% ± 1.6%; Tukey's *p* = 0.970). GDF-11 treatment had no effect on neuronal death at any timepoint following normoxia (Figure 2).

### Increase in Ischemic Neuronal Death by GDF-11 Could Be Blocked by an ALK4/5 Receptor Inhibitor

**Figure 3:**
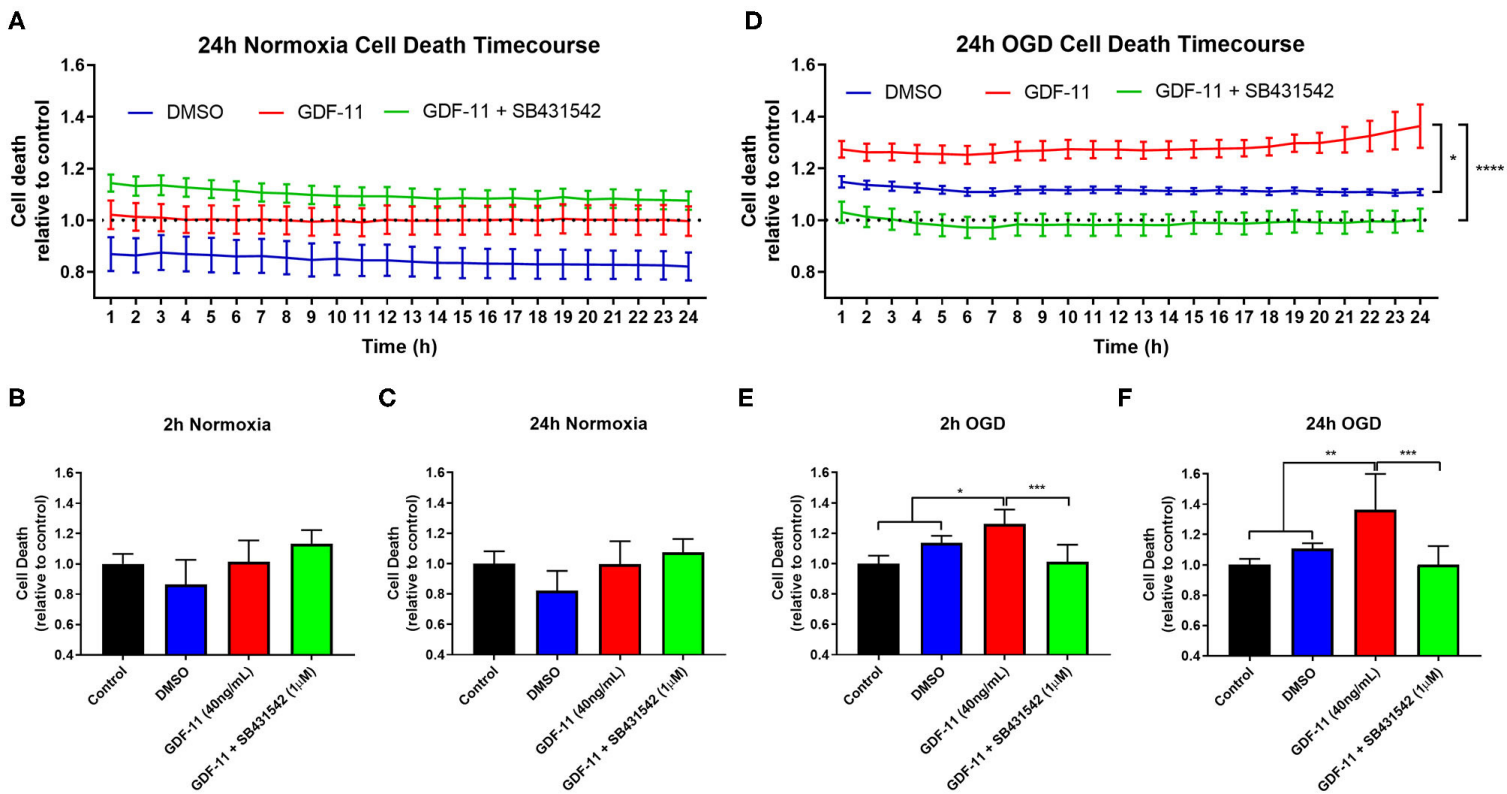
GDF-11 neuronal toxicity during OGD was prevented by a ALK4/5/7 receptor inhibitor. **(A)** GDF-11 +/− SB431542 (ALK4/5/7 receptor inhibitor) had no effect on neuronal death during 24 h of normoxia. **(B)** At 2 h of normoxia, there was no effect of GDF-11 on neuronal death. **(C)** At 24 h of normoxia, GDF-11 did not affect neuronal death. **(D)** GDF-11 enhanced cortical neuronal death throughout 24 h of OGD which was prevented fully by the administration of SB431542. **(E)** At 2 h OGD, GDF-11 increased neuronal death which was reversed by SB431542. **(F)** At 24 h OGD, GDF-11 increased neuronal death which was reversed by SB431542. Cell death was measured by the CellTox™ Green Assay. Data presented as mean ± SEM. *n* = 3 per group except media control which was *n* = 6. One-way ANOVA with Tukey's *post-hoc* test. *p < 0.05; **p < 0.01; ***p < 0.001.

**Figure 4:**
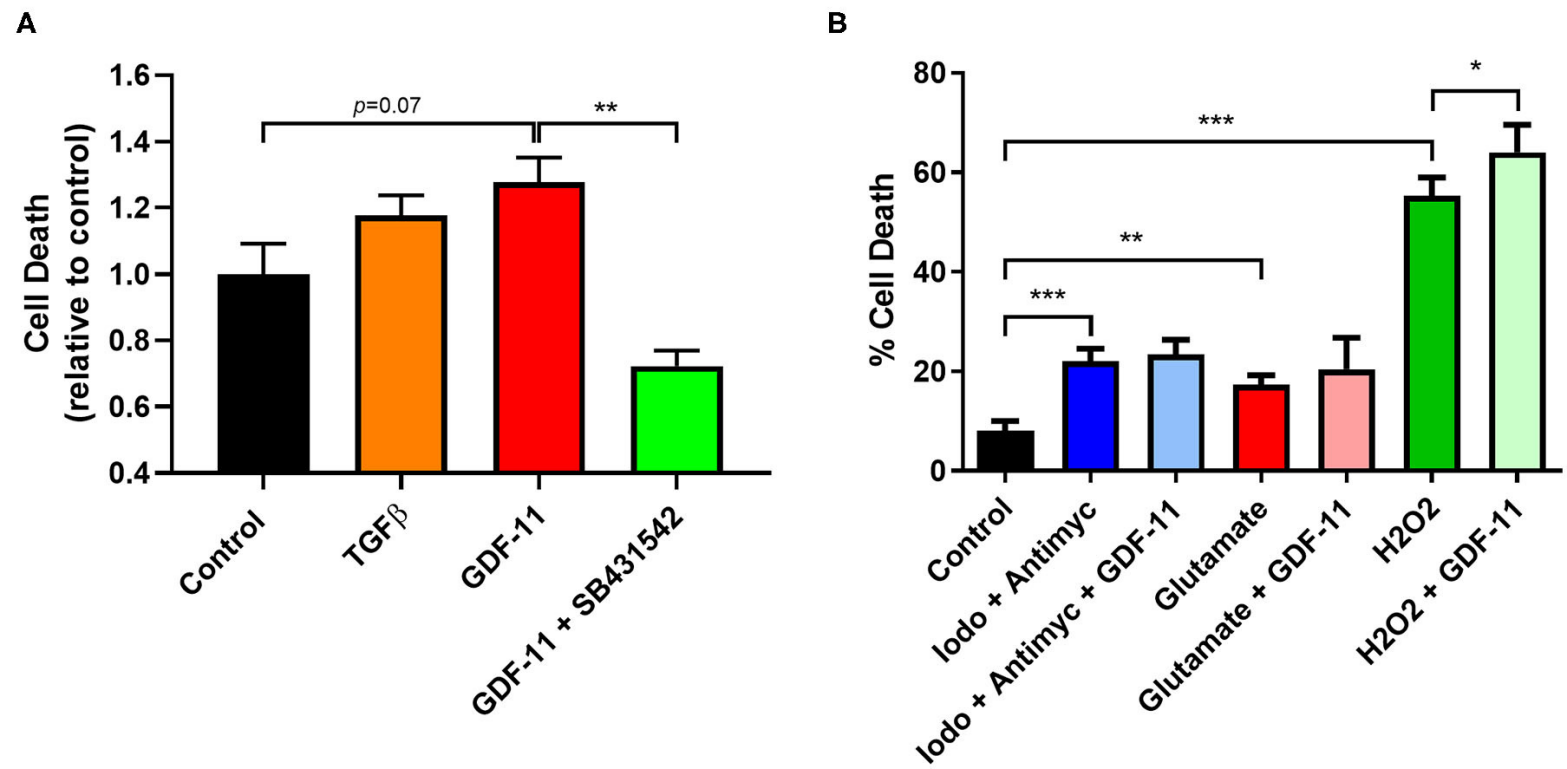
GDF-11 neuronal toxicity occurred under OGD and oxidative stress. **(A)** Neurons were exposed to 2 h OGD followed by 24 h recovery. GDF-11 increased neuronal death while TGFβ did not increase death to the same extent. GDF-11 neurotoxicity was blocked by the ALK4/5/7 inhibitor SB431542. **(B)** Exposure to chemical ischaemia (iodoacetate + antimycin A), excitotoxicity (glutamate) and oxidative stress (H_2_O_2_) for 24 h enhanced neuronal death. GDF-11 increased neuronal death only during oxidative stress (H_2_O_2_) exposure. Data presented as mean ± SEM and were calculated from an LDH assay. *n* = 3–8 per group. One-way ANOVA with Bonferroni *post-hoc* test. *p < 0.05, **p < 0.01, ***p < 0.001.

GDF-11 caused neurotoxicity when administered during OGD and so we sought to identify a mechanism by which this is occurring. Neurons are known to possess a range of TGFβ receptors including ALK4/5 receptors (15, 16) and GDF-11 is known to bind to these receptors (8). We exposed OGD neurons to GDF-11 in the presence or absence of SB431542, an ALK4/5/7 receptor inhibitor (17), and performed real-time monitoring of cell death under OGD or normoxic conditions over a 24 h period (Figures 3A,D). After 2 h OGD (Figure 3E), GDF-11 treatment had increased neuronal death (GDF-11: 1.27 ± 0.03 fold over control; vehicle: 1.15 ± 0.02 over control; Tukey's *p* = 0.0258) which remained raised at 24 h (Figure 3F; GDF-11: 1.36 ± 0.08 fold over control; vehicle: 1.11 ± 0.01 fold over control; Tukey's *p* < 0.0001). Co-treatment of 1 μM SB431542 with GDF-11 prevented GDF-11 neurotoxicity after 2 h OGD (1.03 ± 0.04 fold over control; Tukey's *p* < 0.0001) and 24 h OGD (1.00 ± 0.04 fold over vehicle, Tukey's *p* < 0.0001). No significant effects with GDF-11 nor SB431542 were observed during normoxic conditions (Figures 3B,C). We replicated this experiment using the LDH assay (Figure 4A) and showed that the addition of SB431542 to GDF-11 could restrict cell death (0.72 ± 0.05 fold under control) compared to GDF-11 alone (1.28 ± 0.08, Bonferroni's *p* = 0.0017) following 2 h OGD + 24 h recovery. We also assessed whether administration of TGFβ could replicate the neurotoxicity exhibited by GDF-11 following OGD. Figure 4A demonstrated that while GDF-11 increased cell death compared to control (1.28 ± 0.08 fold over control, Bonferroni's *p* = 0.0743), TGFβ had no significant effect compared to control (1.18 ± 0.06 fold over control, Bonferroni's *p* = 0.349).

### GDF-11 Worsened Neuronal Viability During Oxidative Stress but Not Chemical Ischemia or Excitotoxicity

Other types of stressors occur during an ischemic injury to the brain such as a lack of energy supply, excitotoxicity and oxidative stress all of which can impact on neuronal survival. We modeled these conditions *in vitro* to determine whether GDF-11 could also alter neuronal death under these conditions (Figure 4B). Exposure of neurons for 24 h to a combination of 100 μM iodoacetate (glycolysis inhibitor) and 10 μM antimycin A (mitochondrial respiration inhibitor) to mimic a reduction in energy supply led to increased neuronal death (control: 8.0% ± 1.0%, iodoacetate/antimycin: 22.0% ± 0.9%; Tukey's *p* < 0.0001), but this was not influenced by GDF-11 administration (iodoacetate/antimycin + GDF-11: 23.4% ± 1.0%, Tukey's *p* = 0.9812). Neurons that were subjected to 500 μM glutamate had increased neuronal death (control: 8.0% ± 1.0%, glutamate: 17.2% ± 0.7%; Tukey's *p* = 0.0032), but again this was not influenced by GDF-11 administration (glutamate + GDF-11: 20.5% ± 2.2%, Tukey's *p* = 0.5455). 1 mM hydrogen peroxide indicative of oxidative stress showed a substantial increase in neuronal death (control: 8.0% ± 1.0%, H_2_O_2_: 55.3% ± 1.3%; Tukey's *p* < 0.0001), and interestingly GDF-11 exacerbated neuronal death by hydrogen peroxide (H_2_O_2_ + GDF-11: 64.0% ± 2.0%, Tukey's *p* = 0.0005).

## Discussion

The aging brain is susceptible to a number of different stressors, and the effect of ischemia on the aged brain can cause irreversible damage and subsequent mortality. Recent studies have pointed toward factors that are present in young blood such as GDF-11, that could have potential neuroprotective effects (4, 10, 11). Circulating GDF-11 levels have been shown to decrease with age (4, 6, 19), though this is controversial (5, 20), but this could leave the brain in a susceptible state. Here, we wished to determine whether GDF-11 could have direct protective effects on neurons under simulated ischemic conditions. On the contrary, we discovered that GDF-11 had neurotoxic effects when administered during OGD, and that this neurotoxicity appeared to be due to its activation of the ALK4/5 receptors. GDF-11 neurotoxicity was also observed under oxidative stress conditions. Confining GDF-11 to the bloodstream will help prevent any direct neurotoxic effects of GDF-11 under ischemic conditions.

Under normal conditions, GDF-11 is found in the bloodstream, with a limited amount crossing an intact blood-brain barrier (21). GDF-11 has been shown to have multiple beneficial effects on the vasculature including promotion of angiogenesis, maintaining the blood-brain barrier and providing vascular stability (4, 10, 11, 21). Under *in vivo* stroke conditions, GDF-11 has been shown to reduce infarct volume and improve behavioral outcomes mainly due to both angiogenesis and endothelial cell proliferation (10, 11). However, our evidence suggests that if GDF-11 made it out of the bloodstream and into the brain during ischemia, either through BBB leakage or via some mode of active transport, then it could potentially be harmful to neurons. Therefore, limiting the amount of neuronal exposure to GDF-11 during a stroke could restrict its neurotoxic effects while maintaining their vasculoprotective effects to help improve recovery of the brain.

It is important to note that there was no indication that GDF-11 was neurotoxic under normoxic conditions suggesting that this neurotoxicity was only present when neurons were exposed to ischemia. Likewise, experiments where GDF-11 administration took place prior to or after OGD did not affect neuronal death. This indicates that the susceptibility of neurons to GDF-11-induced neurotoxicity is during ischemia only, but the effects of this can last during the recovery phase. The mechanisms behind why this occurs are unclear, however RNAseq data showed that primary hippocampal neurons subject to OGD increased the expression of both the ALK4 receptor and Smad3 (22). This suggests that the neurotoxic effects of GDF-11 are limited to when the cell becomes stressed, which may augment the activation of the ALK4/Smad3 signaling cascade in neurons.

The type of stress that leads to GDF-11 neurotoxicity is important as there are multiple stressors that can occur during an ischemic episode *in vivo*. Our results suggest that when neurons are oxygen and glucose deprived (limiting energy substrates) and are exposed to oxidative stress (hydrogen peroxide), then GDF-11-induced neurotoxicity ensues, whereas under excitotoxic (excessive glutamate) and chemical ischemia (limiting energy production), GDF-11 neurotoxicity was not observed. However, a recent study showed that under conditions where oxidative stress was increased such as in intracerebral hemorrhage among other pathologies, administration of GDF-11 intraperitoneally promoted production of heme oxygenase-1, an important antioxidant, as well as protecting mitochondrial capacity suggesting indirect effects on neuronal survival (23). This highlights important differences between *in vitro* modeling of neurons and how neurons may react to a stressor *in vivo*, which can be influenced by different cell types in the brain which are not present in single cell type cultures.

GDF-11 is a member of the TGFβ signaling family and has been shown to act at specific TGFβ superfamily receptors including ALK4 and ALK5 (8, 18). Both of these receptors are expressed in neurons and can be activated by Activin A and TGFβ1, respectively (15, 18, 24). Previous studies using Activin A as an agonist at ALK4 receptors and TGFβ1 as an agonist at ALK5 receptors have shown neuroprotective properties in *in vivo* models of stroke (9, 25). While our data showing direct GDF-11 neurotoxicity under ischemic conditions is in contrast to the neuroprotective effects of these agonists, the exposure of TGFβ1 to neurons subjected chronically to glutamate led to neurotoxicity (26). Our data suggest that GDF-11 neurotoxicity was due to its action on the ALK4 receptor. TGFβ administration (which only activates ALK5 receptors) did not increase neuronal death to the same extent as GDF-11. This is further supported by the evidence that the ALK4/5/7 inhibitor SB431542 could block the neurotoxic effects of GDF-11 during OGD. Moreover, RNAseq analysis of hippocampal neurons following OGD and recovery has demonstrated that there was an increase in both ALK4 receptor expression and Smad3 signaling following OGD (22). Therefore, GDF-11 administration may put greater stress on the Smad signaling cascade through ALK4 activation leading to detrimental outcomes to neurons.

Exposure of hydrogen peroxide to neurons led to exacerbation of neuronal death by GDF-11 which suggests that free radical formation may be an important mechanism of cell death by GDF-11. OGD of neuronal cultures can directly lead to reactive oxygen species generation, which can be blocked by free radical scavengers (27). Interestingly, a traditional pro-survival factor, neurotrophin-3, unexpectedly showed damaging effects on neurons following OGD due to increased reactive oxygen species formation (28). Furthermore, there is evidence in other disease conditions that activation of TGFβ and Smad signaling can lead to reactive oxygen species formation (29). Confirmation with pharmacological studies, using agents such as SB431542, would be valuable to confirm that these effects are mediated through the ALK4/5 receptors. Overall, there appears to be a link between the TGFβ signaling cascade and free radical formation, which could mediate GDF-11 neurotoxicity under ischemic and oxidative stress conditions, but further experiments investigating mitochondrial changes by GDF-11 under these conditions are needed to confirm these effects.

There are some limitations to this study. One limitation was the utilization of only one concentration of GDF-11 in the neurotoxicity experiments (40 ng/mL). We showed that GDF-11 at a range of concentrations (4–400 ng/mL) had no effects on neuronal viability under normoxic conditions, and so we carefully chose a concentration within this range that had exhibited effects in previous studies (4) and had a concentration above the half maximal effective concentration (EC_50_) to activate Smad signaling in other cell types (30). However, we cannot rule out the possibility that effects, beneficial or neurotoxic, could be observed at other concentrations of GDF-11 following OGD. Another limitation is that the isolated neuronal culture method may not reflect the effects of GDF-11 in the complex brain environment. However, determining direct effects of GDF-11 on neurons *in vivo* or in *ex vivo* brain slices is difficult, particularly due to the strong vascular effects that GDF-11 are known to have (4, 10, 11, 21), meaning that the viability of neurons will be influenced by the effects of GDF-11 on other cell types in experiments using these methodologies.

These results reveal for the first time that GDF-11 is neurotoxic to primary neurons in the acute phase of simulated stroke through primarily ALK4 receptor signaling. Therefore, limiting GDF-11 access to the brain during ischemia could prevent its neurotoxicity. Future studies will further characterize the neurotoxic vs. protective properties of GDF-11 in the brain and whether the protection of the brain is solely mediated through the vascular system.

## Data Availability Statement

The raw data supporting the conclusions of this article will be made available by the authors upon request.

## Author Contributions

BS, GH, AN, YC, and AB conceived and designed the study. BS, GH, ZA, TL, and NK carried out the experiments. BS, ZA, TL, and KM analyzed the data. BS wrote the manuscript. All authors edited and approved the final version.

## Conflict of Interest

AB is a senior medical science advisor and co-founder of Brainomix, a company that develops electronic ASPECTS (e-ASPECTS), an automated method to evaluate ASPECTS in stroke patients. The remaining authors declare that the research was conducted in the absence of any commercial or financial relationships that could be construed as a potential conflict of interest.
